# Brain Functional Interaction of Acupuncture Effects in Diarrhea-Dominant Irritable Bowel Syndrome

**DOI:** 10.3389/fnins.2020.608688

**Published:** 2020-12-15

**Authors:** Kai Ma, Yongkang Liu, Wei Shao, Jianhua Sun, Jing Li, Xiaokun Fang, Jing Li, Zhongqiu Wang, Daoqiang Zhang

**Affiliations:** ^1^MIIT Key Laboratory of Pattern Analysis and Machine Intelligence, College of Computer Science and Technology, Nanjing University of Aeronautics and Astronautics, Nanjing, China; ^2^Department of Radiology, The Affiliated Hospital of Nanjing University of Chinese Medicine, Nanjing, China; ^3^Department of Acupuncture and Rehabilitation, The Affiliated Hospital of Nanjing University of Chinese Medicine, Nanjing, China; ^4^Department of Acupuncture and Moxibustion, Nanjing Hospital of Chinese Medicine affiliated to Nanjing University of Chinese Medicine, Nanjing, China

**Keywords:** fMRI, complex network, irritable bowel syndrome, brain function, acupuncture stimulation

## Abstract

Acupuncture is a traditional Chinese medicine treatment that has widely been used to modulate gastrointestinal dysfunction caused by irritable bowel syndrome (IBS) and to alleviate the resulting pain. Recent studies have shown that gastrointestinal dysfunction caused by IBS is associated with dysregulation of the brain's central and peripheral nervous system, while functional magnetic resonance imaging (fMRI) helps explore functional abnormality of the brain. However, previous studies rarely used fMRI to study the correlations between brain functional connection, interaction, or segregation (e.g., network degree and clustering coefficient) and acupuncture stimulation in IBS. To bridge this knowledge gap, we study the changed brain functional connection, interaction, and segregation before and after acupuncture stimulation for diarrhea-dominant IBS (IBS-D) with the help of complex network methods based on fMRI. Our results indicate that the abnormal functional connections (FCs) in the right hippocampus, right superior occipital gyrus, left lingual gyrus, left middle occipital gyrus, and the cerebellum, and abnormal network degree in right middle occipital gyrus, where normal controls are significantly different from IBS-D patients, are improved after acupuncture stimulation. These changed FCs and the network degree before and after acupuncture stimulation have significant correlations with the changed clinical information including IBS symptom severity score (*r* = −0.54, *p* = 0.0065) and IBS quality of life (*r* = 0.426, *p* = 0.038). We conclude that the changes of the brain functional connection, interaction, and segregation in the hippocampus, middle and superior occipital gyrus, cerebellum, and the lingual gyrus may be related to acupuncture stimulation. The abnormal functional connection, interaction, and segregation in IBS-D may be improved after acupuncture stimulation.

## 1. Introduction

Irritable bowel syndrome (IBS) is a kind of intestinal disorder characterized by abdominal pain, abdominal distension, changed defecation habits, and stool characteristics, accompanying gastrointestinal dysfunction and biochemical abnormalities (Mayer, 2008). The onset age of these diseases is usually between 20 and 50 and the incidence in females is higher than that for males (Lovell and Ford, 2012). According to different characteristics of human stool, IBS could be divided into four clinical subtypes, including the diarrhea-dominant type (called IBS-D), constipation type, mixed type, and the amorphous type (Holtmann et al., 2016). The etiology and pathogenesis of IBS remain unclear until now, and multiple reasons could result in differences from clinical types involving abnormal gastrointestinal motility (Drossman et al., 1982; Tillisch et al., 2011), visceral paresthesia (Kiraly et al., 2006), dysregulation of bidirectional brain-gut interactions (Mayer and Tillisch, 2011), inflammation (Tornblom et al., 2002; Holtmann et al., 2016), and psychosocial state (Evans et al., 1996; Whitehead et al., 2002). In existing studies, various functional mechanisms and biopsychosocial models (Tanaka et al., 2011) related to clinical types have been put forward to detect the pathogenesis of IBS. Recent studies have shown that gastrointestinal dysfunction caused by IBS is associated with the dysregulation of the brain's central and peripheral nervous system (Omalley, 2016; Wang et al., 2017) and may be altered by psychological factors such as stress, anxiety, and depression (Ke et al., 2015). Few of these studies make the effort to utilize functional interaction and segregation to investigate the brain dysfunction mechanism of IBS and those modulated by related treatments such as acupuncture stimulation.

Functional magnetic resonance imaging (fMRI) is a very effective noninvasive technique to study brain function mechanisms. It has widely been used to study the mechanisms of the brain's central and peripheral nervous system included in brain-gut interactions and the association between gastrointestinal dysfunction and brain functional abnormities (Mayer et al., 2006). With the aid of fMRI, the specific brain regional activities in IBS patients that differ from those of normal controls (NC) and the correlations between clinical information and brain dysfunction affected by IBS could be identified (Dong et al., 2014; Qi et al., 2016). While most of the existing fMRI-based studies aimed at revealing the abnormal brain function mechanisms caused by IBS in local brain regions, few attempts have been made to use fMRI to study brain function mechanisms modulated by acupuncture stimulation.

As a traditional Chinese medicine treatment, acupuncture has been widely used in China for more than 3,000 years and plays an important role in the treatment of chronic diseases in contemporary medicine (Lee et al., 2008; Cheng et al., 2018). For the treatment of these chronic diseases, pain relief, and improvement of body function are two main application directions of acupuncture stimulation. Recent studies have shown that acupuncture could modulate hypothalamus-limbic systems resulting in higher efficiency and stronger small-world properties in brain function of normal controls (Hennig and Lacour, 2000; Pei et al., 2014). As a specific type of IBS, diarrhea-dominant IBS (IBS-D) has the symptoms of abdominal pain and intestinal discomfort (Whitehead et al., 1980; Mayer, 2008) and brings great trouble to people's daily life. The relevant research showed that the gastrointestinal dysfunction caused by IBS might be associated with the disorders of hypothalamus-limbic nervous systems (Aenck, 2006; Qin et al., 2017). Therefore, we think that acupuncture stimulation could be utilized to relieve pain and modulate related nervous systems to improve the gastrointestinal dysfunction of IBS-D patients. However, few studies tried to explore the brain function mechanisms related to gastrointestinal dysfunction caused by IBS-D and those modulated by acupuncture stimulation combining brain functional interaction and segregation.

The complex network methods based on graph theory could be used to investigate brain functional interaction and segregation mechanisms (Bullmore and Sporns, 2012; Wu et al., 2016). Hence, we utilize functional connections and network characteristics including degree, clustering coefficient, and local efficiency to detect brain functional connection, interaction, and segregation related to gastrointestinal dysfunction caused by IBS-D and acupuncture stimulation. We also investigate the correlations between the changed brain functional connection, interaction, or segregation and clinical information. Here, we hypothesize that the functional connections and network characteristics of IBS-D patients without acupuncture stimulation have significant differences with those of IBS-D patients with acupuncture stimulation. The changed functional connections and network characteristics have the correlations with the changed clinical information including IBS symptom severity score (IBS-SSS), IBS quality of life (IBS-QOL), and the Hamilton anxiety scale (HAMA). First, we statistically analyze the functional connections, degree, clustering coefficient, and local efficiency between IBS-D patients without acupuncture stimulation and normal controls, IBS-D patients with acupuncture stimulation and those without, and IBS-D patients with acupuncture stimulation and NC. We picked out the abnormal functional connections, degrees, and clustering coefficients where there are significant differences among NC, IBS-D patients with acupuncture stimulation and those without. We then detect the correlations between the changed functional connections, degree, clustering coefficient, and changed clinical information.

## 2. Methods and Materials

### 2.1. Participants

The prospective research was approved by the local institutional review board and was conducted in compliance with the Health Insurance Portability and Accountability Act of 1996. All right-handed subjects with normal mental ability participated in this study were provided informed consent before functional MRI scanning, acupuncture stimulation and neurological evaluations. We assessed the severity of disease in IBS-D patients based on two indicators, i.e., IBS Symptom Severity Score (IBS-SSS) (Betz et al., 2013) and IBS Quality of Life (IBS-QOL) (Drossman et al., 2000). Besides, the Hamilton Anxiety Scale (HAMA) (Snaith et al., 1982) was also used to evaluate the anxiety degree between IBS-D patients and normal controls.

We recruited 43 IBS-D (diarrhea-dominant Irritable Bowel Syndrome) patients who received a diagnosis, and their brains were first scanned at the Jiangsu Province Hospital of Chinese Medicine. Twenty-nine of the patients underwent acupuncture stimulation and received the second scans at the Jiangsu Province Hospital of Chinese Medicine. Five patients who received acupuncture stimulation were excluded from the study due to inconsistent scan parameters. We also recruited 25 age-matched normal controls, and two of them were excluded from this study due to inconsistent scanning parameters. Therefore, 24 IBS-D patients and 23 normal controls were finally included in this study. Inclusion criteria for IBS-D patients were as follows: (a) age between 18 and 55 years old, (b) IBS-SSS above 75 on baseline, (c) no drug treatment within 2 weeks, (d) no history of acupuncture stimulation within 3 months related to IBS, and (e) no participation in other clinical research projects. Exclusion criteria were listed as follows: (a) other IBS subtypes, (b) organic gastrointestinal disease or history of gastrointestinal surgery, (c) other confounding medical conditions (such as diabetes, hyperthyroidism, mental illness, and other chronic pains), (d) serious heart, liver, and kidney illness, (e) pregnant or lactating women, (f) vivo magnetic implants (such as iron, or with cochlear implants, vascular clips, or with pacemaker), and (g) metal sensitivity or afraid of needle. The demographic and clinical information of subjects are given in Table 1.

**Table 1:** Demographic and clinical information of subjects.

**Parameter**	**Control group**	**IBSbs group**	**IBS1st group**	***P*-value**
	**(*n* = 23)**	**(*n* = 24)**	**(*n* = 24)**	
Age	41.96(12.7)	40.17 (10.6)	40.17 (10.6)	0.603^+^
Gender (M/F)	14/9	17/7	17/7	0.547^*^
Years of education	17.83 (3.8)	18.17 (2.4)	18.17 (2.4)	0.718^+^
IBS-SSS	–	245.5 (100)	91.9 (49)	<0.0001^+^
IBS-QOL	–	132.46 (33.1)	150.33 (13.6)	0.02^+^
HAMA	2.48 (2.9)	15.25 (7.9)	8.17 (2.9)	<0.0001^†^

*Data were means, with standard errors in parentheses. IBS-SSS, IBS Symptom Severity Score; IBS-QOL, IBS quality of life. HAMA, Hamilton anxiety scale; IBSbs group were IBS-D patients who did not undergo acupuncture stimulation. IBS1st group were IBS-D patients undergoing acupuncture stimulation. P < 0.05 was considered to indicate a significant difference*.

+ *According to the two-sample t-test*.

* *According to the χ^2^ test*.

† *According to one-way analysis of variance (Bonferroni correction)*.

### 2.2. Acupuncture Protocol

We provided comfort, diet, lifestyle review and consultation to IBS-D patients. We needed to help IBS-D patients relieve stress and reduce avoidance behavior and established a positive doctor-patient relationship. Moreover, we also needed to help IBS-D patients establish regular eating times, adequate liquid intake, and adequate physical activity. Quality of life, activities of daily living, personality characteristics, recent life stress events, anxiety, and depression were reviewed. Health lifestyle education was provided.

**Figure 1:**
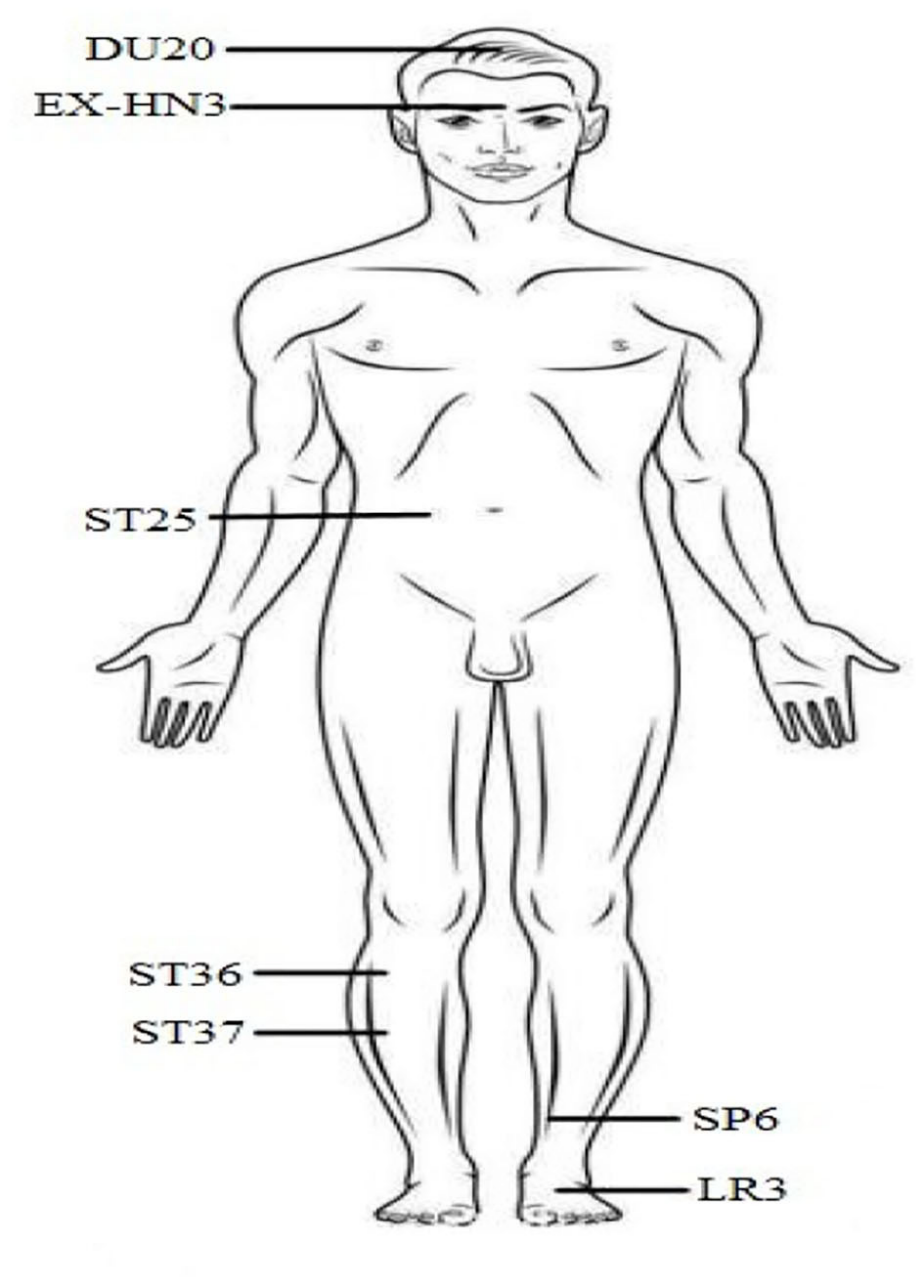
Seven acupuncture points (acupoints) on the skin for acupuncture intervention in this study, including bilateral Bai Hui (DU20), Yin Tang (EX-HN3), Tai Chong (LR3), Zu Sanli (ST36), San Yinjiao (SP6), Tian Shu (ST25), and Shang Juxu (ST37).

The IBS-D patients received acupuncture stimulations at seven acupuncture points (acupoints) and these points were determined according to the point localization method issued by the World Health Organization (WHO) (Lim, 2010). These acupoints included bilateral Bai Hui (DU20), Yin Tang (EX-HN3), Tai Chong (LR3), Zu Sanli (ST36), San Yinjiao (SP6), Tian Shu (ST25), and Shang Juxu (ST37) (see Figure 1). The tools of acupuncture stimulation were sterile acupuncture needles with a diameter of 0.3 mm and a length of 40 mm. After cleaning the skin with a tincture of iodine and alcohol, these needles were inserted into seven acupuncture points in each patient. The acupuncture protocol followed the standard permissible depth of insertion for each acupoint. Specifically, DU20 and EX-HN3 were punctured obliquely 0.5–0.8 cun (a unit of length, 1 cun = 1/3 decimeter) and 0.2–0.3 cun into the skin, respectively. Three acupoints (i.e., ST36, ST37, and SP6) were punctured 1 cun into the skin. Additionally, ST25 and LR3 were punctured 1–1.5 and 0.5 cun, respectively.

Acupuncturists with more than 6 years of clinical experience performed acupuncture manipulation moderately, to achieve an optimal acupuncture response named Deqi (a sensation of soreness, numbness, fullness, and heaviness, and/or adjacent muscle twitching) (Zhang et al., 2018). The acupuncture manipulation included lifting, thrusting and twirling. For each patient, the needles inserted into seven acupoints needed to be held for 30 min before being pulled out in each session. During the acupuncture period, needles were manipulated twice every 10 min with intermittent stimulation, and intermittent stimulation lasted for 10 s. For each patient, this acupuncture stimulation was performed every other day (up to three times a week) for 6 consecutive weeks.

### 2.3. fMRI Data Acquisition

IBS-D patients were first scanned when they came to the hospital for diagnosis. During this period, they did not receive any acupuncture stimulation. Soon afterwards they were treated with acupuncture for 6 consecutive weeks. After undergoing all acupuncture stimulations, these patients were scanned again. The subjects lay supine with the head snugly fixed by straps and foam pads to minimize head movement. During the resting-state session, the subjects were instructed to keep as still as possible and not to think systematically.

The fMRI scans of subjects were performed in the Jiangsu Province Hospital of Chinese Medicine using a SIEMENS Veno 3-Tesla scanner with 18-channel phased-array head coil. The functional images were obtained using an EPI sequence with the following parameters: Volume numbers = 210, 42 axial slices for each data volume, Slice thickness = 3.5 mm, in-plane resolution = 128 × 128, TR = 2,310 ms, TE = 21 ms, flip angle = 90°, FOV = 224 × 224*mm*. In addition, a T1-weighted sagittal three-dimensional magnetization-prepared rapid gradient echo (MPRAGE) sequence was acquired, covering the entire brain: 176 slices, TR = 2,300 ms, TE = 2.19 ms, slice thickness = 1 mm, flip angle = 9°.

### 2.4. Functional MR Imaging Data Preprocessing

All acquired data sets were preprocessed with DPARSF (Yan and Zang, 2010), which is a pipeline data analysis tool combining statistical parametric mapping (SPM) (http://www.fil.ion.ucl.ac.uk/spm) and resting-state fMRI data analysis toolkit (REST) (http://www.restfmri.net) for resting-state fMRI. DICOM data was transformed to the neuroimaging informatics technology initiative (NIfTI) file format. The first 10 time points from each subject's data were discarded for signal equilibrium and to enable the subjects to adapt to the scanning noise. Slice-timing correction was applied using the middle slice as the reference frame. Head motion correction was executed to adjust the time series of images in order to correct the brain in the same position in every image. Friston 24 motion parameters, white matter (WM), and cerebrospinal fluid signals were regressed. The T1-weighted MR image of each subject is segmented into WM, GM, and cerebrospinal fluid (CSF). In order to remove the possible influence of CSF and WM in the fMRI time series, the GM tissue of each subject is utilized to mask their corresponding fMRI data. Functional images were spatially normalized into the standard Montreal Neurological Institute (MNI) space using echo-planar imaging (EPI) template. DPARSF smoothed the data using a Gaussian kernel with a full-width at half-maximum (FWHM) of 4 mm. The systematic drift or trend was removed by using the linear model.

### 2.5. Construction of Functional Brain Connectivity Network

The whole-brain cortical and subcortical structures were subdivided into 116 brain regions for each subject, based on the Automated Anatomical Labeling (AAL) atlas (Peer et al., 2017; Zhuo et al., 2018). Each brain region was regarded as a region-of-interest (ROI), and the mean rs-fMRI time series was calculated by averaging the blood oxygen level-dependent (BOLD) signals among all voxels within each specific ROI. The linear correlation between mean time series of a pair of ROIs was then calculated to measure the functional connectivity between the paired ROIs. Accordingly, a 116 × 116 fully-connected weighted functional network was constructed for each subject, where each node denoted a particular ROI and the edge weight was the Pearson correlation coefficient corresponding to a pair of nodes/ROIs. Then, Fisher's z transformation was applied to the elements of the functional network to improve the normality of Pearson correlation coefficients.

In this work, we treat the edge weights as the strength of functional connectome between pairs of brain regions. Previous studies have indicated that self-connections and negative connections (such as functional anti-correlation) need to be removed because weak and non-significant links represent spurious connections (Rubinov and Sporns, 2010; Li et al., 2011). Therefore, we only utilized positive functional connections (Liao et al., 2014; Finn et al., 2015) to obtain binarized brain networks of all subjects and then use them to calculate the brain functional network properties (Rubinov and Sporns, 2010; Kruschwitz et al., 2018). Briefly speaking, in a weight network, if its edge weight is larger than zero, this edge is set to one, otherwise, it is zero. Using this sparsity approach, we obtain a binarized brain network for each subject.

### 2.6. Statistical Analysis

The diversities of demographic characteristics (i.e., age and education years) were evaluated using the two-sample *t*-test. The diversities of clinical scores (i.e., IBS-SSS and IBS-QOL) were evaluated using the paired-samples *t*-test. The measure of HAMA was evaluated using one-way analysis of variance (ANOVA). The proportions of subject sex (female to male ratio) between normal controls and IBS-D patients were analyzed using the χ^2^ test. In addition to the first scanning, those IBS-D patients who underwent acupuncture stimulation were scanned again after 6 weeks. To detect the effect of acupuncture stimulation on brain function mechanisms, we needed to compare not only the differences from IBS-D patients before and after acupuncture stimulation, but also the differences between IBS-D patients and normal controls. When comparing three groups of data, we applied one-way ANOVA to compare their diversities of brain function mechanisms. When comparing IBS-D patients before and after acupuncture stimulation, we used paired-samples *t*-test to detect the differences of brain function mechanisms. The pairwise comparisons were performed between normal controls and IBS-D patients with two-sample *t*-test. Bonferroni correction was applied to correct multiple-comparisons. Pearson correlation coefficient was utilized to analyze the correlation between brain function mechanisms and clinical information.

### 2.7. Network Characteristic

Network static characteristics, which described the mechanisms of brain functional interaction and segregation with graph theory, were used to understand and explore the brain disorders in order to reveal their neural mechanism. Here, we used node degree, node clustering coefficient, and local efficiency to investigate the acupuncture effects in functional interaction and segregation mechanisms of brain regions.

#### 2.7.1. Degree

Degree is a common measure of functional interaction, which has a straightforward neurobiological interpretation. Nodes with high degrees interact functionally with many other nodes in the network [36]. Degree of a node *i* is defined as the number of links connected to a node, which is calculated as follows:

(1) $$\begin{array}{l} k_{i}=\underset{j\in N}{\sum }a_{ij} \end{array}$$

Where, *a*_*ij*_ is the edge between node *i* and node *j*.

#### 2.7.2. Clustering Coefficient

Clustering coefficient is a measure of functional segregation, which reflects the prevalence of clustered connectivity around individual brain regions. The ratio of the actual number of edges *E*_*i*_ to the total number of possible edges $C_{k_{i}}^{2}$ between *k*_*i*_ neighbors of node *v*_*i*_ is defined as the clustering coefficient of node *v*_*i*_ (see Equation 2).

(2) $$\begin{array}{l} C_{i}=\frac{E_{i}}{C_{k_{i}}^{2}} \end{array}$$

#### 2.7.3. Local Efficiency

Local efficiency is also a measure of functional segregation, which reflects the information transmission capability of brain region. To determine the nodal (regional) characteristics of networks, we computed the local efficiency, as follows:

(3) $$\begin{array}{l} E_{node}\left(i\right)=\frac{1}{N}\underset{i\neq j\in G}{\sum }\frac{1}{L_{ij}^{\prime }} \end{array}$$

Where $L_{ij}^{\prime }$ is the shortest path length that is a measure of integration between nodes *i* and *j* in subnetwork *G*′. *E*_*node*_(*i*) is utilized to measure the mean shortest path length between a given node and all other nodes in subnetwork *G*′.

### 2.8. Classification Verification

In order to verify whether the cured and activated functional connections still existed in the brain networks of the other IBS-D patients with acupuncture stimulation and those without, we collected a new batch of fMRI datasets including eight IBS-D patients and eight normal controls. The subject information of new IBS-D patients and normal controls were matched. These eight IBS-D patients were scanned before and after acupuncture stimulation with same parameters in section of fMRI data acquisition in section 2. Eight normal controls were also scanned with same parameters. We utilized support vector machine (SVM) classifier with a radial basis function (RBF) kernel to classify NC, IBSbs and IBS1st in these cured and activated functional connections. We utilized the old fMRI datasets as the training set and the new dataset as the test set. The SVM classifier used in the present study was provided by LIBSVM toolbox (Chung and Lin, 2011).

## 3. Results

The complex network methods used to investigate brain function mechanisms based on functional interaction and segregation included functional connection and network characteristic (Bullmore and Sporns, 2012). We first divided all subjects into three subgroups: (1) normal controls (NC), (2) IBS-D baseline patients without acupuncture stimulation (called IBSbs), and (3) IBS-D patients with acupuncture stimulation (denoted as IBS1st). Before statistical analysis in group comparison, we investigated the sparsity of brain functional network among IBSbs, IBS1st, and NC. By performing a group comparison among the three groups (i.e., NC, IBSbs, and IBS1st) based on complex network methods, we detected disorder functional connections, cured, and activated brain functional connections, and network characteristics in brain networks of three groups (i.e., IBS1st, IBSbs, and NC). Here, the disorder functional connections represented that the functional connections of IBSbs are significantly different from those of NC. The term “cured” denotes that IBSbs have changed functional connections and network characteristics compared to NC, these changed connections and characteristics still existed between IBSbs and IBS1st, but these connections and characteristics of IBS1st returned to the normal level (i.e., mean connection strength in NC group) after acupuncture stimulation (see **Figures 3**, **4**). Besides, the term “activated” denotes that IBS-D patients without acupuncture stimulation have similar functional connections and network characteristics compared to NC, i.e., the functional connections and network characteristics of IBSbs and NC were from the same probability distribution. These connections and characteristics were changed compared to NC and IBSbs (decrease below or increase over the normal level) after acupuncture stimulation (see **Figure 6**).

### 3.1. Sparsity Analysis

**Figure 2:**
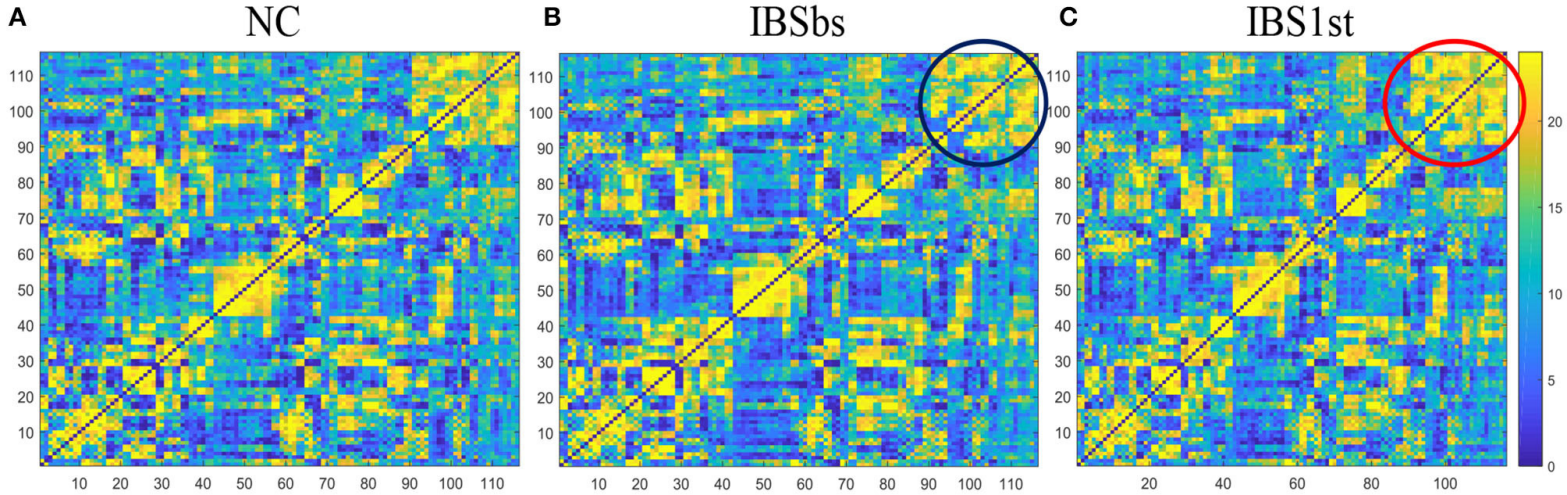
Frequency connection matrices. **(A)** NC is normal control. **(B)** IBSbs: IBS-D baseline patients without acupuncture stimulation. **(C)** IBS1st: IBS-D patients with acupuncture stimulation. The color in the figure represents the frequency of edge connection between pairwise brain regions.

Before analyzing brain functional connections, we investigated the sparsity of brain network matrices among IBS, IBS1st, and NC. In our experiment, the number of IBS-D patients (*n* = 24) was similar to the number of normal controls (*n* = 23). We used the frequency of edge connection to measure the connection probability between pairwise brain regions. If m subjects had the edge connection between pairwise brain regions, the frequency of this edge connection was m. We plotted the frequency connection matrices for IBS, IBS1st, and NC (see Figure 2).

We calculated the number of unconnected edges in frequency connection matrices in Figure 2. We found that the number of unconnected edges in NC was the same as IBSbs in the whole matrices and was larger than the number in IBS1st. In other words, the matrix sparsity of NC was the same as IBSbs but was larger than IBS1st. The sub-matrix formed by the cerebellum in IBSbs—see the black circle in Figure 2B–was sparser than NC. After acupuncture stimulation, this sub-matrix in IBS1st became dense–see the red circle in Figure 2C. We inferred that the sparse connections in the sub-matrix formed by the cerebellum in IBSbs might be related to IBS-D. Furthermore, acupuncture stimulation might have some effects on this sub-matrix.

### 3.2. Disorder Functional Connections

**Figure 3:**
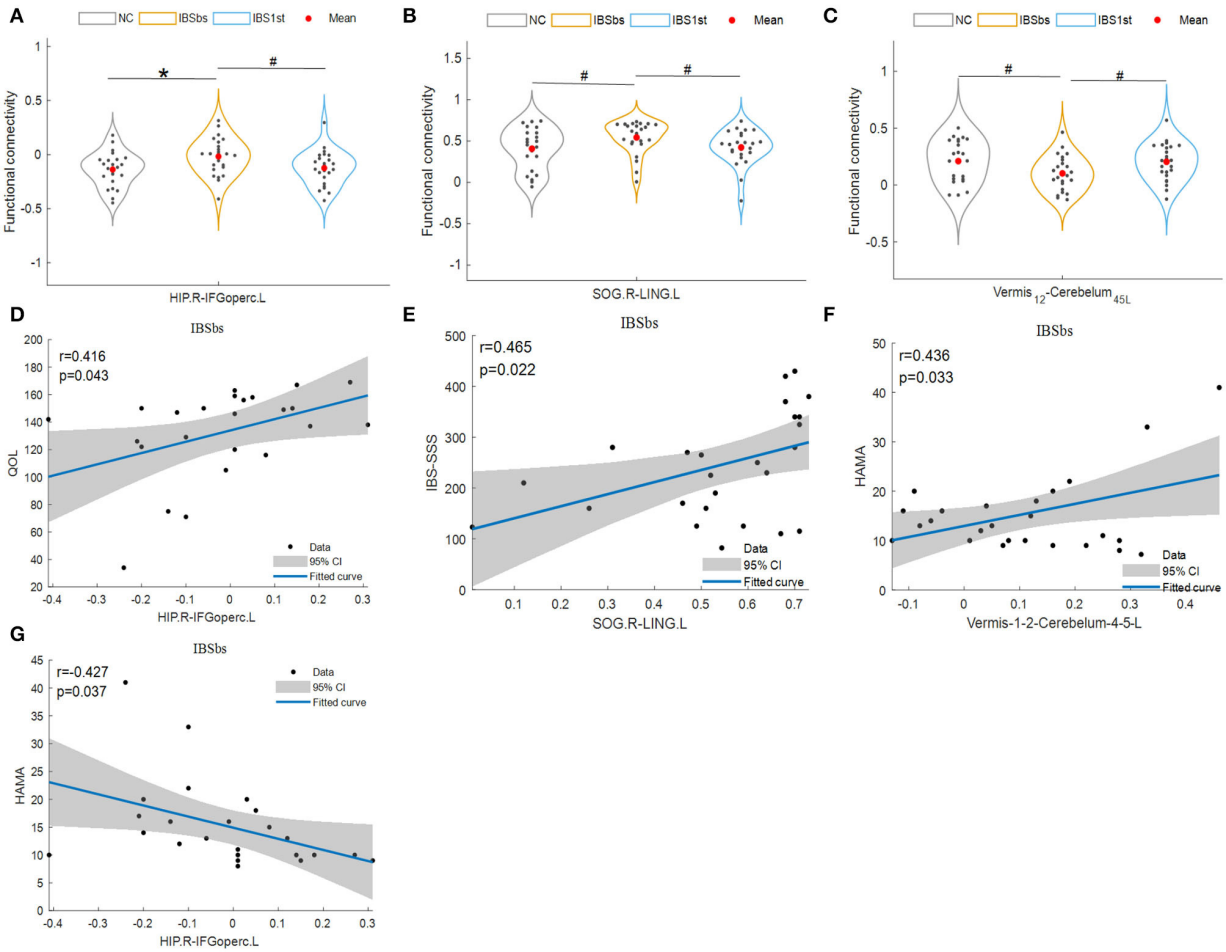
Disorder functional connections. HIP.R, right hippocampus; IFGoperc.L, left inferior frontal gyrus, opercular part; SOG.R, right superior occipital gyrus; LING.L, left lingual gyrus. Vermis12 is the part in cerebellar regions. Cerebelum45.L, left cerebelum45 which is a part of cerebellar regions; IBS-SSS, IBS Symptom Severity Score; HAMA, Hamilton Anxiety Scale; QOL, IBS Quality of Life. The mark * denotes *p* < 0.05 (Bonferroni correction). The mark # indicates *p* < 0.05 (uncorrected) with the significance level of 0.05. NC is normal control. IBSbs: IBS-D baseline patients without acupuncture stimulation. IBS1st: IBS-D patients with acupuncture stimulation. The x axis in **(A–C)** represents the location of each subject (e.g., patient or normal control). The x axis in **(D–G)** represents the functional connections between pairwise brain regions.

**Figure 4:**
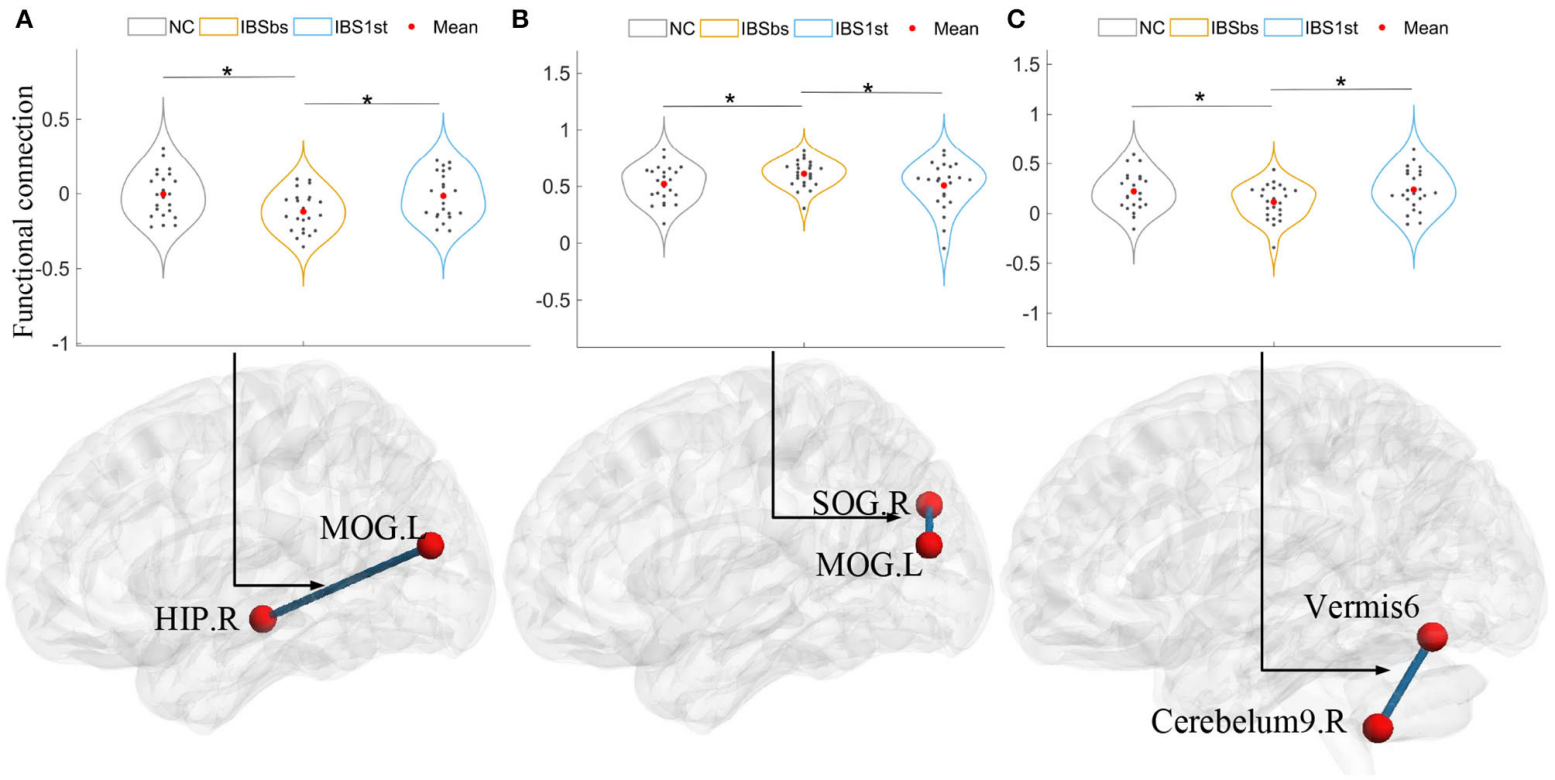
Cured functional connections. HIP.R, right hippocampus; MOG.L, left middle occipital gyrus; SOG.R, right superior occipital gyrus; Cerebelum9.R, right cerebelum9. Vermis6 and right cerebelum9 are parts of the cerebellums. The mark * denotes *p* < 0.05 (Bonferroni correction). NC, normal control; IBSbs, IBS-D baseline patients without acupuncture stimulation; IBS1st, IBS-D patients with acupuncture stimulation. The x axis in **(A–C)** represents the location of each subject (e.g., patient or normal control).

IBSbs did not have differences, except for IBS-D with NC. Hence, we hypothesized that the disorder functional connections might be caused by IBS-D. We first analyzed the disorder functional connections which might be related to the gastrointestinal dysfunction (see Figures 3, 4). The disorder functional connections included functional connections between the right hippocampus (HIP.R) and the left inferior frontal gyrus, the opercular part (IFGoperc.L) (Figure 3A), right superior occipital gyrus (SOG.R), and the left lingual gyrus (LING.L) (Figure 3B), vermis12 and the left cerebelum45 (cerebelum45.L) (Figure 3C), HIP.R and MOG.L (Figure 4A), SOG.R and the left middle occipital gyrus (MOG.L) (Figure 4B), and vermis6 and the right cerebelum9 (Cerebelum9.R) (Figure 4C).

We used Pearson correlation to calculate the correlations and found that the disorder functional connections among the right HIP, left IFGoperc, right SOG, left LING, vermis12, and the left cerebelum45 were significantly related to the clinical information including QOL, IBS-SSS, and HAMA. The functional connections between the right HIP and the left IFGoperc were positively related to QOL (*r* = 0.416, *p* = 0.043, Figure 3D), and negatively related to HAMA (*r* = −0.427, *p* = 0.037, Figure 3G). The functional connections between the right SOG and the left LING were positively related to IBS-SSS (*r* = 0.465, *p* = 0.022, Figure 3E). The functional connections between vermis12 and the left cerebelum45 were positively related to HAMA (*r* = 0.436, *p* = 0.033, Figure 3F). Here, we speculated that the disorder functional connections among the right HIP, left IFGoperc, right SOG, left LING, vermis12, and the left cerebelum45 might be related to gastrointestinal dysfunction caused by IBS-D.

### 3.3. Cured and Activated Functional Connections

In Figure 4, we plotted the “cured” functional connections between the pairwise brain regions including the right hippocampus (HIP.R), left middle occipital gyrus (MOG.L), right superior occipital gyrus (SOG.R), the vermis6, and the right cerebelum9 (Cerebelum9.R). The functional connections of IBSbs between the right hippocampus and left middle occipital gyrus (Figure 4A), and the right cerebelum9 and vermis6 (Figure 4C) were significantly lower than those of NC. After acupuncture stimulation, the functional connections of IBS1st were significantly higher than those of IBSbs but returned to the normal level. The functional connections of IBSbs between the left middle occipital gyrus and the right superior occipital gyrus were significantly higher than those of NC—these connections returned to the normal level after acupuncture stimulation (Figure 4B).

**Figure 5:**
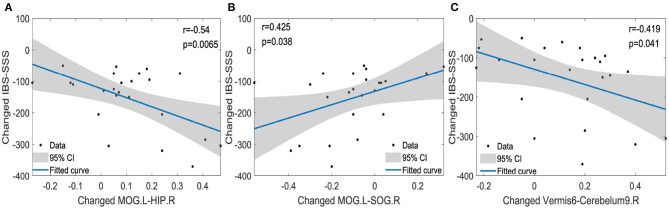
The correlations between changed functional connections and changed clinical information. IBS-SSS, IBS Symptom Severity Score; HIP.R, right hippocampus; MOG.L, left middle occipital gyrus; SOG.R, right superior occipital gyrus; Cerebelum9.R, right cerebelum9. Vermis6 and right cerebelum9 are cerebellums. CI: confidence interval (two-sided test). The x axis in **(A–C)** represents the changed functional connections between pairwise brain regions (e.g., changed functional connections between MOG.L and HIP.R).

We detected the correlations calculated by Pearson correlation between changed and cured functional connections and changed clinical information including IBS-SSS, QOL, and HAMA. The changed functional connections were functional connections of IBS1st minus the functional connections of IBSbs. The changed clinical information was the clinical information of IBS1st minus the clinical information of IBSbs. We found that the changed functional connections between the left MOG and the right HIP were negatively correlated to the changed IBS-SSS (*r* = −0.54, *p* = 0.0065; see Figure 5A). The changed functional connections between the left MOG and the right SOG were positively correlated to the changed IBS-SSS (*r* = 0.425, *p* = 0.038; see Figure 5B). The changed functional connections between the vermis6 and the right cerebelum9 were negatively correlated to the changed IBS-SSS (*r* = −0.419, *p* = 0.041; see Figure 5C).

**Figure 6:**
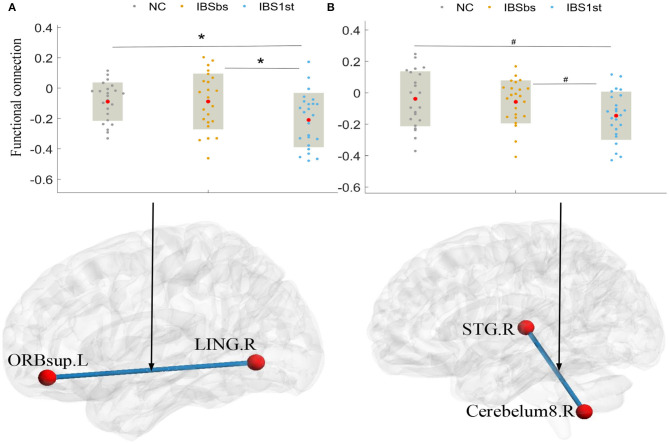
Activated functional connections. The mark * denotes *p* < 0.05 (Bonferroni correction). The mark # indicates *p* < 0.05 (uncorrected) with the significance level of 0.05. ORBsup.L: left superior frontal gyrus and orbital part. LING.R, right lingual gyrus; STG.R, right superior temporal gyrus; Cerebelum8.R, right cerebelum8; NC, normal control; IBSbs, IBS-D baseline patients without acupuncture stimulation; IBS1st, IBS-D patients with acupuncture stimulation. The x axis in **(A,B)** represents the location of each subject (e.g., patient or normal control).

Additionally, we found that the acupuncture stimulation activated the related functional connections between the left superior frontal gyrus, orbital part (ORBsup.L) and the right lingual gyrus (LING.R) and between the right superior temporal gyrus (STG.R) and the right cerebelum8 (cerebelum8.R). After acupuncture stimulation, the functional connections of IBS1st between the left ORBsup and right LING and between the right STG and right cerebelum8 were significantly lower than those of NC and IBSbs (see Figures 6A,B).

**Figure 7:**
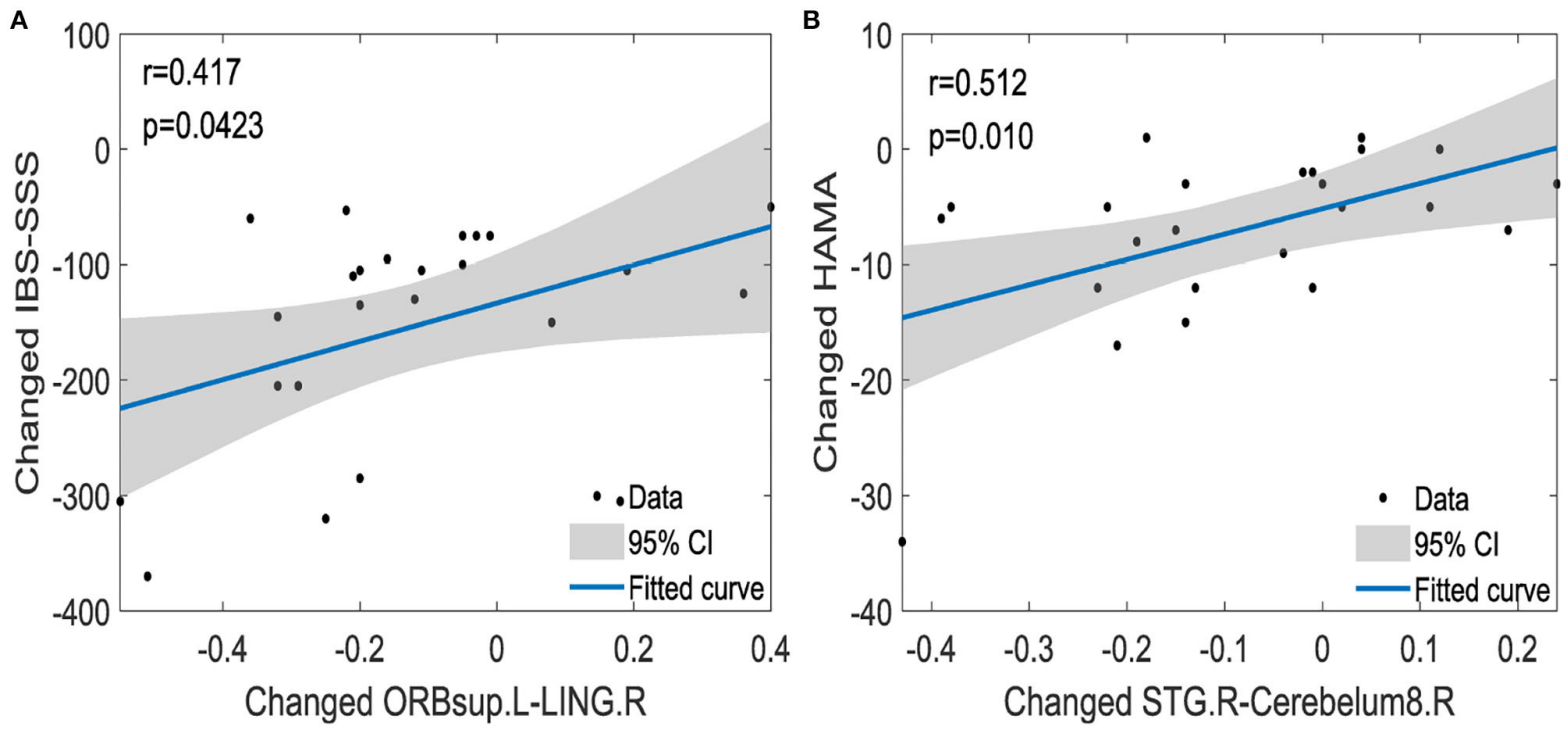
The correlations between changed functional connections and changed clinical information [IBS-SSS **(A)**, HAMA **(B)**]. IBS-SSS, IBS Symptom Severity Score; HAMA, Hamilton Anxiety Scale; ORBsup.L, left superior frontal gyrus and orbital part; LING.R, right lingual gyrus; STG.R, right superior temporal gyrus; Cerebelum8.R, right cerebelum8; CI, confidence interval (two-sided test). The x axis in **(A,B)** represents the changed functional connections between pairwise brain regions (e.g., changed functional connections between ORBsup.L and LING.R).

We analyzed the correlations calculated by Pearson correlation between changed clinical information and changed functional connections among the left ORBsup, right LING, right STG, and the right cerebelum8. The changed functional connections between the left ORBsup and right LING were positively correlated to the changed IBS-SSS (*r* = 0.417, *p* = 0.0423) (see Figure 7A). The changed functional connections between the right STG and right cerebelum8 were positively correlated to changed HAMA (*r* = 0.512, *p* = 0.01; see Figure 7B).

### 3.4. Functional Interaction and Segregation

**Figure 8:**
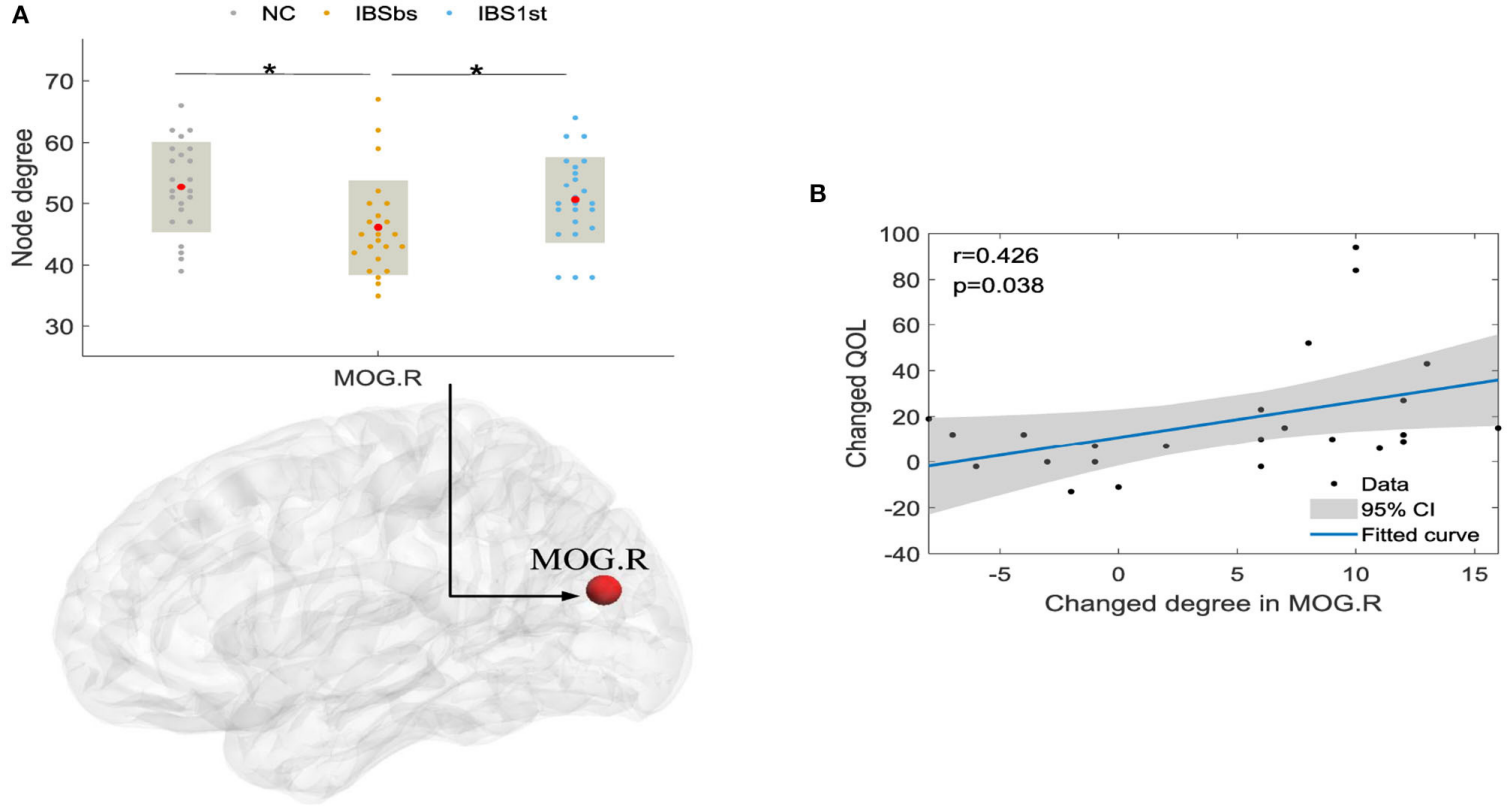
The degree in right MOG **(A)** and correlation between changed QOL and changed degree in MOG.R **(B)**. MOG.R, right middle occipital gyrus. The mark * denotes *p* < 0.05 (Bonferroni correction). CI, confidence interval (two-sided test). QOL, IBS Quality of Life; NC, normal control; IBSbs, IBS-D baseline patients without acupuncture stimulation; IBS1st, IBS-D patients with acupuncture stimulation. The x axis in **(A)** represents the location of each subject (e.g., patient or normal control).

We also investigated the effect of acupuncture stimulation in functional interaction and segregation with network properties including node degree, clustering coefficient, and local efficiency. As shown in Figure 8A, we found that the degrees of IBSbs in right middle occipital gyrus (MOG.R) were significantly lower than those of NC. After acupuncture stimulation, the degrees of IBS1st in the right MOG were significantly higher than those of IBSbs but were similar to those of NC.

We analyzed the correlations calculated by Pearson correlation between the changed degrees and the changed QOL. The changed degrees were the degrees of IBS1st minus the degrees of IBSbs. The changed QOL were the QOL of IBS1st minus the QOL of IBSbs. We found that the changed degrees in the right MOG were positively correlated to the changed QOL (*r* = 0.426, *p* = 0.038; see Figure 8B).

**Figure 9:**
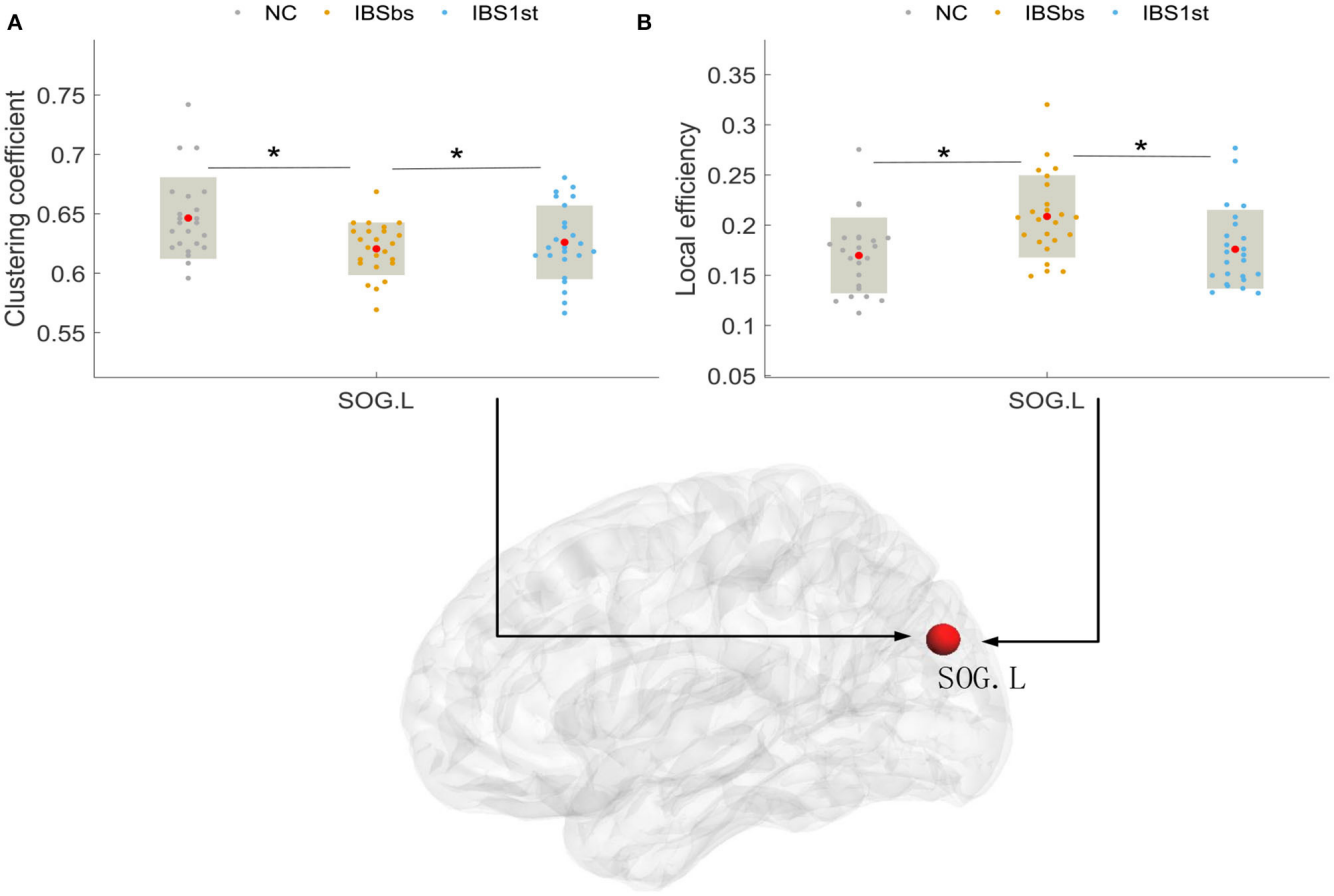
The clustering coefficient **(A)** and local efficiency **(B)** in left superior occipital gyrus (SOG.L). The mark * denotes *p* < 0.05 (Bonferroni correction). NC, normal control; IBSbs, IBS-D baseline patients without acupuncture stimulation; IBS1st, IBS-D patients with acupuncture stimulation. The x axis in **(A,B)** represents the location of each subject (e.g., patient or normal control).

Lastly, we detected the clustering coefficient and local efficiency in the left superior occipital gyrus (SOG.L). We found that the clustering coefficients of IBSbs in the left SOG were significantly lower than those of NC. The clustering coefficients of IBS1st in the left SOG were significantly higher than those of IBSbs, but were similar to those of NC (see Figure 9A). The local efficiency of IBSbs in the left SOG were significantly higher than those of NC. The local efficiencies of IBS1st in the left SOG were significantly lower than those of IBSbs, but were similar to those of NC; see Figure 9B. We inferred that the acupuncture stimulation affected the functional interaction and segregation in the right MOG and the left SOG.

### 3.5. Classification Verification

**Figure 10:**
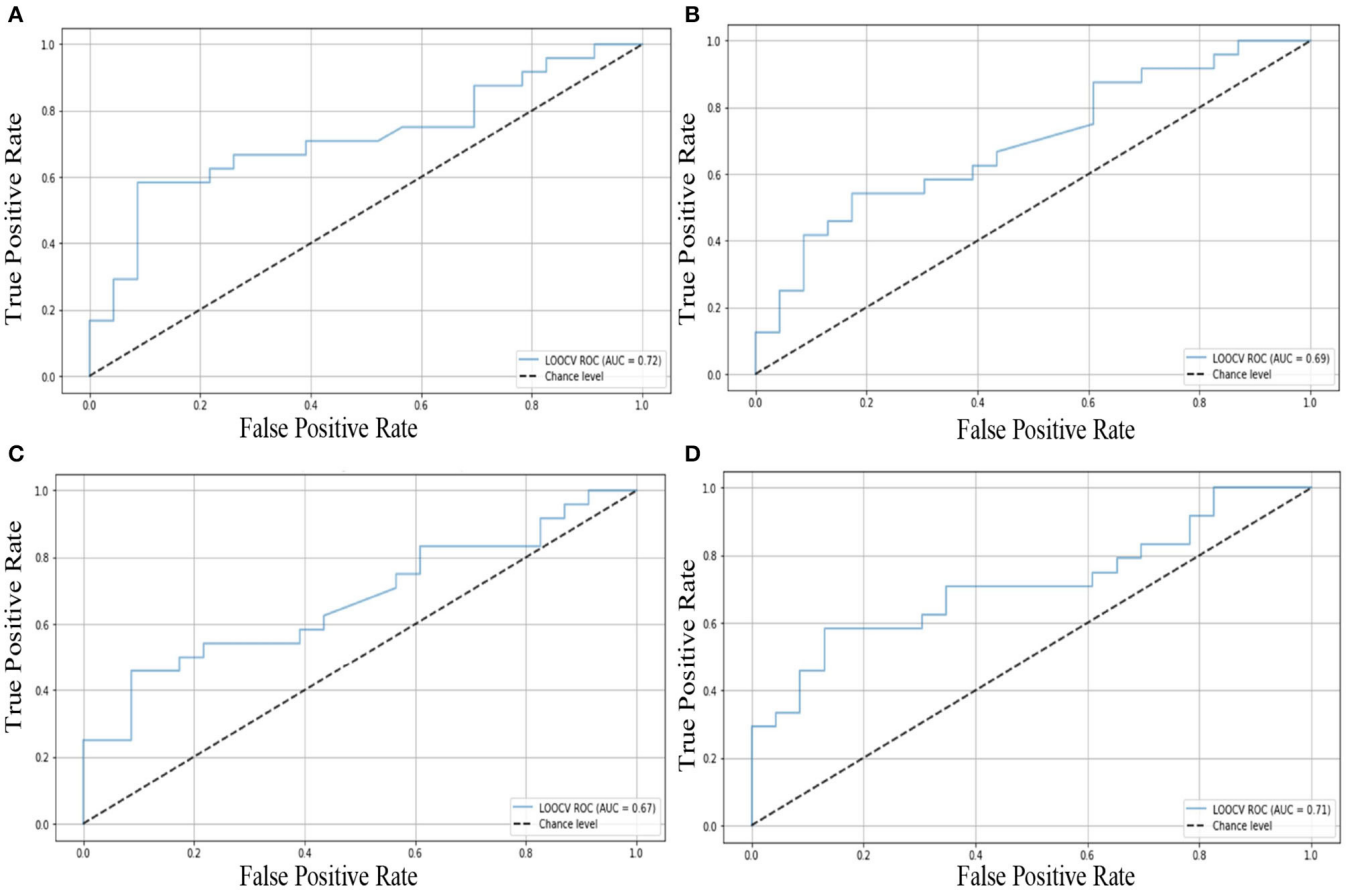
Classification verification in cured and activated functional connections. **(A)** The ROC in cured functional connections between NC and IBSbs. **(B)** The ROC in cured functional connections between IBSbs and IBS1st. **(C)** The ROC in activated functional connections between IBSbs and IBS1st. **(D)** The ROC in activated functional connections between NC and IBS1st.

In order to verify whether the cured and activated functional connections still existed in the brain networks of new IBS-D patients with acupuncture stimulation and those without, as elucidated by the statistical analysis, we collected a new batch of data. We utilized a machine learning-based classifier (i.e., support vector machine, SVM) to classify NC, IBSbs, and IBS1st based on the cured and activated functional connections in the new dataset. We trained the model on the old data set and used the new data as the test set. The classification results showed that these significantly discriminated features in cured and activated functional connections could effectively distinguish NC from the IBSbs, IBSbs from IBS1st, and NC from IBS1st; see the receiver operating characteristic (ROC) curve in Figure 10. The classification accuracy in cured functional connections between NC and IBSbs is 73.2%; the ROC curve can be seen in Figure 10A. The classification accuracy in cured functional connections between IBSbs and IBS1st is 70.8%; the ROC curve can be seen in Figure 10B. The classification accuracy in activated functional connections between IBSbs and IBS1st is 69.4%; the ROC curve can be seen in Figure 10C. The classification accuracy in activated functional connections between NC and IBS1st is 72.6%; the ROC curve can be seen in Figure 10D. The functional connections between the right hippocampus and left middle occipital gyrus, between the left middle occipital gyrus and right superior occipital gyrus, and between the right cerebelum9 and vermis6 could, therefore, be regarded as the functional connections cured after acupuncture stimulation. The functional connections between the left superior frontal gyrus orbital part and the right lingual gyrus and between the right superior temporal gyrus and the right cerebelum8 could be regarded as the functional connections activated after acupuncture stimulation.

## 4. Discussion

We used the complex network methods based on fMRI to explore the mechanism of brain functional connection, interaction, and segregation of IBS-D patients before and after acupuncture stimulation. We found that the disorder functional connections among the right hippocampus (Ying et al., 2018), left inferior frontal gyrus, the opercular part, the right superior occipital gyrus, left lingual gyrus (Jiaofen et al., 2019), and the cerebellum were related to clinical information including IBS quality of life, the IBS symptom severity score, and the Hamilton anxiety scale. We inferred that these functional connections might be related to gastrointestinal dysfunction caused by IBS-D. After acupuncture stimulation, the changed functional connections between the right hippocampus and left middle occipital gyrus (Liu et al., 2010), between the left middle occipital gyrus and right superior occipital gyrus, and between the vermis6 and right cerebelum9 were related to the IBS symptom severity score. We inferred that acupuncture stimulation affected these functional connections. Moreover, we also found that acupuncture stimulation affected functional interaction (i.e., degree) (Cecilia et al., 2018) and segregation (i.e., clustering coefficient and local efficiency) in the right middle occipital gyrus and the left superior occipital gyrus (Batalle et al., 2012; Kartun-Giles and Bianconi, 2019).

### 4.1. Acupuncture Effects in Brain Functional Connectivity

It was observed that there was a close relationship between acupuncture effects and the functional connections (Cao et al., 2020) related to the right hippocampus (He et al., 2018), the left middle occipital gyrus (Zhang et al., 2015), the right superior occipital gyrus, the vermis6, the right cerebelum9, the left superior frontal gyrus-orbital part, the right lingual gyrus and the right superior temporal gyrus. The functional connections of IBSbs between the right hippocampus (Seminowicz et al., 2010; Niddam et al., 2011; Afifa et al., 2018) and left middle occipital gyrus (Figure 4A), between the left middle occipital gyrus and right superior occipital gyrus (Figure 4B), and between the right cerebelum9 and vermis6 (Figure 4C) were significantly different from those of NC (Weng et al., 2017), which could be regarded as the abnormal functional connections caused by gastrointestinal dysfunction. After acupuncture stimulation, these abnormal functional connections returned to the normal level and there were no significant differences between NC and IBS1st in these connections. We speculated that the acupuncture stimulation cured these abnormal functional connections. We found that these cured functional connections were related to the IBS Symptom Severity Score. The functional connections of IBSbs between the right hippocampus and left middle occipital gyrus and between the right cerebelum9 and vermis6 were increased after acupuncture stimulation. The increased functional connections were negatively correlated to the changed IBS-SSS (*r* = −0.54, *p* = 0.0065, *r* = −0.419, *p* = 0.041). The functional connections of IBSbs between the left middle occipital gyrus and the right superior occipital gyrus were decreased after acupuncture stimulation. The decreased functional connections were positively correlated to the changed IBS-SSS (*r* = 0.425, *p* = 0.038). These results further demonstrated that acupuncture stimulation cured the abnormal functional connections between the right hippocampus and left middle occipital gyrus, between the left middle occipital gyrus and right superior occipital gyrus, and between the right cerebelum9 and vermis6.

We also found that the acupuncture stimulation improved the IBS symptom severity and anxiety of IBS-D patients by activating the related functional connections. The acupuncture stimulation negatively activated the functional connections between the left superior frontal gyrus orbital part and right lingual gyrus and between the right superior temporal gyrus (Song et al., 2006) and right cerebelum8, which were decreased compared with those of IBSbs. The decreased functional connections between the left superior frontal gyrus orbital part and the right lingual gyrus were positively related to the changed IBS Symptom Severity Score (*r* = 0.417, *p* = 0.0423). The decreased functional connections between the right superior temporal gyrus and the right cerebelum8 were positively related to the changed Hamilton Anxiety Scale (*r* = 0.512, *p* = 0.01).

### 4.2. Acupuncture Effects on Local Brain Interaction and Segregation

After analyzing the functional connections affected by acupuncture stimulation, we detected the brain functional interaction and segregation (Ma et al., 2020). We found that the global brain functional interaction and segregation were not affected by acupuncture stimulation. We then investigated the local brain functional interaction and segregation. We found that the degrees in the right middle occipital gyrus were affected by acupuncture stimulation. After acupuncture stimulation, the degrees in the right middle occipital gyrus were increased compared with those of IBSbs and returned to the normal level. The increased degrees in the right middle occipital gyrus were positively related to IBS Quality of Life (*r* = 0.426, *p* = 0.038) (Atluri et al., 2013). We speculated that the acupuncture stimulation improved the quality of life of IBS patients by positively activating the functional interactions between the right middle occipital gyrus and its contiguous brain regions. The abnormal clustering coefficients and local efficiencies in the left superior occipital gyrus were affected by acupuncture stimulation. The clustering coefficient and local efficiency reflected the brain functional segregation. We therefore speculated the acupuncture stimulation improved the abnormal brain functional segregation mechanism in the left superior occipital gyrus (Li, 2013).

### 4.3. Classification Verification

We used a machine learning-based classifier (i.e., support vector machine, SVM) (Chung and Lin, 2011) to classify normal controls, IBS-D patients with acupuncture stimulation, and those without based on the cured and activated functional connections in the new dataset. Our classification results indicated that these cured and activated functional connections could effectively classify normal controls, IBS-D patients with acupuncture stimulation, and those without in the new dataset. The cured functional connections included functional connections among the right hippocampus, left middle occipital gyrus, right superior occipital gyrus, vermis6, and the right cerebelum9. The activated functional connections included functional connections among the left superior frontal gyrus-orbital part, right lingual gyrus, right superior temporal gyrus, and the right cerebelum8. We inferred that these functional connections could be regarded as the potential biomarkers. In the diagnosis of brain diseases, we could also use these functional connections as features to diagnose IBS-D patients.

### 4.4. Limitations

There were still some limitations in our research. Our research did not compare patients with acupuncture stimulation to normal controls undergoing acupuncture stimulation. In the future, we will take this comparative experiment into account. In the construction of the functional brain connectivity network, we only used the Automated Anatomical Labeling (AAL) atlas. We did not utilize other atlases to verify our findings. In the future, we will choose different atlases to verify our findings. In our analysis, we only utilized positive functional connections to obtain binarized brain networks of all subjects. It might ignore the effect of the negative functional connections. Furthermore, there are many different sparsity methods which could be used to construct the binarized brain networks. The different sparsity methods may result in different sparsity results. In the future, we will utilize different sparsity methods to verify our findings.

## 5. Conclusion

Using complex network methods, we have uncovered more useful brain function mechanisms which may be affected by IBS-D and acupuncture stimulation. Most of these function mechanisms are related to clinical information. Our results indicate that the abnormal functional connections in the right hippocampus, right superior occipital gyrus, left lingual gyrus, left middle occipital gyrus, and the cerebellum and an abnormal network degree in the right middle occipital gyrus, have no difference to those of normal controls after acupuncture stimulation. The changed functional connections and network degree before and after acupuncture stimulation have significant correlations with the changed clinical information, including the IBS symptom severity score and the IBS quality of life. We infer that acupuncture stimulation in Bai Hui (DU20), Yin Tang (EX-HN3), Tai Chong (LR3), Zu Sanli (ST36), San Yinjiao (SP6), Tian Shu (ST25), and Shang Juxu (ST37) may affect the functional connections and local brain functional interaction and segregation in the hippocampus, superior occipital gyrus, lingual gyrus, middle occipital gyrus, and the cerebellum. These brain regions are related to the brain's central and peripheral nervous system. Our research is therefore helpful for investigating the brain functional mechanism modulated by acupuncture stimulation in diarrhea-dominant irritable bowel syndrome.

## Data Availability Statement

The original contributions presented in the study are included in the article/supplementary material, further inquiries can be directed to the corresponding author/s.

## Ethics Statement

The studies involving human participants were reviewed and approved by Ethics Committee of Affiliated Hospital of Nanjing University of Chinese Medicine (Jiangsu Province Hospital of Chinese Medicine). The participants provided their written informed consent to participate in this study.

## Author Contributions

KM, YL, ZW, and DZ designed the whole study. JL (5th author), XF, and JL (7th author) performed the research of acupuncture stimulation and evaluated the acupuncture effect. YL scanned the subjects and collected their data. KM and WS wrote the paper. All authors contributed to the article and approved the submitted version.

## Conflict of Interest

The authors declare that the research was conducted in the absence of any commercial or financial relationships that could be construed as a potential conflict of interest.
